# Genome-wide association study in a lettuce core collection from 811 accessions reveals genetic loci for anthocyanin accumulation and cultivar development

**DOI:** 10.1093/hr/uhaf258

**Published:** 2025-09-24

**Authors:** Guotao Huo, Haibin Wei, Shuping He, Guojun Ge, Lei Wang, Guangliu Xu, Yan Huang, Yiwen Zhou, Xiao Yang, Zhenzhen Li, Yingyan Han, Shiwei Wei, Lijun Luo

**Affiliations:** Shanghai Agrobiological Gene Center, Shanghai 201106, China; Shanghai Agrobiological Gene Center, Shanghai 201106, China; Shanghai Agrobiological Gene Center, Shanghai 201106, China; Shanghai Agrobiological Gene Center, Shanghai 201106, China; Shanghai Agrobiological Gene Center, Shanghai 201106, China; Shanghai Agrobiological Gene Center, Shanghai 201106, China; Shanghai Agrobiological Gene Center, Shanghai 201106, China; Shanghai Agrobiological Gene Center, Shanghai 201106, China; Chengdu National Agricultural Science and Technology Center, Institute of Urban Agriculture, Chinese Academy of Agricultural Sciences, Chengdu 610213, China; Shanghai Extension and Service Center of Agriculture Technology, Shanghai 210103, China; Beijing Key Laboratory of New Technology in Agricultural Application, National Demonstration Center for Experimental Plant Production Education, College of Plant Science and Technology, Beijing University of Agriculture, Beijing 102206, China; Shanghai Agrobiological Gene Center, Shanghai 201106, China; Shanghai Agrobiological Gene Center, Shanghai 201106, China

## Abstract

Lettuce (*Lactuca sativa*) is a globally cultivated leafy vegetable with leafy morphology critically influencing consumer preference and market value. Despite the agronomic importance of leaf traits, the genetic basis underlying their diversity remains poorly characterized. To address this, we resequenced 811 accessions collected from major lettuce production areas as well as the relative wild species, and developed a publicly accessible core collection of 268 accessions that captures 99.4% of the total genetic variation. Phenotypic evaluation of 16 leaf morphological traits across two growing seasons identified significant correlations, including negative associations between plant width and anthocyanin content, and positive correlations between apical margin incision and multiple traits. Population structure analysis revealed frequent introgression events from looseleaf type into domesticated varieties (butterhead, crisphead, romaine, and stem lettuce), highlighting dynamic gene flow during breeding. Genome-wide association studies (GWAS) pinpointed 13 robust quantitative trait loci (QTLs) and candidate genes regulating leaf morphology, including a validated anthocyanin biosynthesis regulator (*ANS*). Notably, we pinpointed the causal gene genotypes responsible for leaf anthocyanin coloration. Leveraging these findings, we successfully aggregated favorable alleles through genomic design breeding to develop a novel high-anthocyanin variety binfen5 with desirable leaf morphology. This integrative approach demonstrates the value of core germplasms and genomic tools for accelerating lettuce improvement.

## Introduction

Lettuce (*Lactuca sativa*) is a globally cultivated leafy vegetable with significant economic and nutritional value. Its consumption has increased dramatically worldwide since the late 20th century [1]. The cultivated lettuce originated from a single domestication event ~4000 BC and appeared in China ~1700 years ago through the Silk Road [2, 3]. The domesticated lettuces primarily present a morphological diversity of its leaves, such as leaf shape, anthocyanin content, and margin architecture. Its agronomic importance directly impacts consumer preference, marketability, and adaptability to varying environmental conditions. The genetic mechanisms governing these traits remain poorly resolved, limiting targeted breeding efforts.

Germplasm collections serve as cornerstones of biodiversity conservation, particularly in preserving genetic resources vital for food security, breeding applications, and research on origin and evolution [2–4]. The diversity of lettuce is a result of many factors, its global spread, long cultivation history, and local eating habits. Stem lettuce, known as woju and wosun in China, has become an alternative to traditional lettuce varieties. Youmaicai is also a popular lettuce in China, used for leaf and stem in cooking. To date, youmaicai lack evidence for their domestication origin. Previous studies on multi-omics in LettuceGDB and LettuceDB integrating rich germplasm resources have contributed to the economic value and academic significance for lettuce [4, 5]. Population structure analysis using single nucleotide polymorphism (SNP) markers revealed five and six subpopulations in 148 and 298 homozygous lettuce lines, respectively [6, 7]. The low marker density limited the separation of closely related varieties, leading to imprecise conclusion [8]. Phylogenetic relationships deduced from RNA sequencing of 240 lettuce accessions suggested that all lettuce cultivars originated from the same ancestor undergoing a single domestication [2]. The single domestication history of cultivated lettuce was validated by genome resequencing of 445 *Lactuca* accessions [3]. Thus, germplasm collections of lettuce are essential to gene mining and utilization for crop improvement through the storing allelic diversity.

Lettuce research has lagged in leveraging modern genomic tools to systematically characterize its genetic diversity or define a representative core collection, a resource critical for efficient gene discovery and preservation. Core germplasm collections are essential for maintaining genetic diversity and ensuring the success of crop breeding programs. A core germplasm is a representative subset of a larger germplasm collection, designed to capture the maximum amount of genetic diversity with minimal redundancy [9]. These collections serve as valuable genetic resources for improving desirable agronomic traits in crops, including disease resistance, drought tolerance, and yield potential. The advent of high-throughput sequencing and molecular marker technologies has revolutionized the ability to assess genetic variation across large germplasm collections [10, 11]. Moreover, recent advances in genome-wide association studies (GWAS) and genomic selection offer unprecedented opportunities to link phenotypic variation with underlying genetic loci, as exemplified by successful identifying novel genes in the anthocyanin biosynthesis in lettuce [2]. The accumulation of flavonoids, particularly anthocyanins, is a key biochemical process responsible for red, purple, or blue pigmentation in plant tissues [12]. It is a multifaceted trait that enhances leaf quality through stress resilience, nutritional value, and marketability. Yet, such approaches remain underutilized in lettuce, particularly for dissecting leaf morphology, a complex trait influenced by polygenic interactions and environmental plasticity. Therefore, given the vast size of many germplasm collections, selecting a manageable subset that captures the key genetic variation becomes essential for maximizing breeding efficiency. By integrating genomic data with phenotypic data, it is possible to identify core germplasms that not only represent genetic diversity but also exhibit desirable phenotypic traits necessary for crop improvement [13, 14]. For horticultural crops like lettuce, where breeding programs often aim to address complex traits such as bolting time, leaf morphology, and disease resistance, the efficient use of genetic resources is critical.

To better understand the patterns of genetic variability and genomic design breeding strategies, we developed a core collection of 268 lettuce accessions capturing >99.4% of genetic variation from 811 germplasm, providing a publicly accessible collection for genomics-assisted breeding of lettuce. The core set, including modern cultivars, traditional landraces, and wild species, was whole-genome sequenced up to ~5x genomic coverage and characterized genetic diversity, population structure, phylogenetic relationships, linkage disequilibrium, and population differentiation. For the genetic dissection of leaf morphological traits, we performed the GWAS to identify genetic loci and select candidate genes associated with phenotypic variation of lettuce leaf. The adaptive genetic variations based on human selection are critical for their value in crop improvement. Based on the GWAS results, we performed a hybridization to generate a high-anthocyanin variety binfen5 for both visual appeal and health benefits. These results provide important genomic resources for evolutionary biology and molecular breeding of lettuce, and stronger foundation for research on genetic improvement.

## Results

### Development of the core lettuce germplasm collection

**Figure 1:**
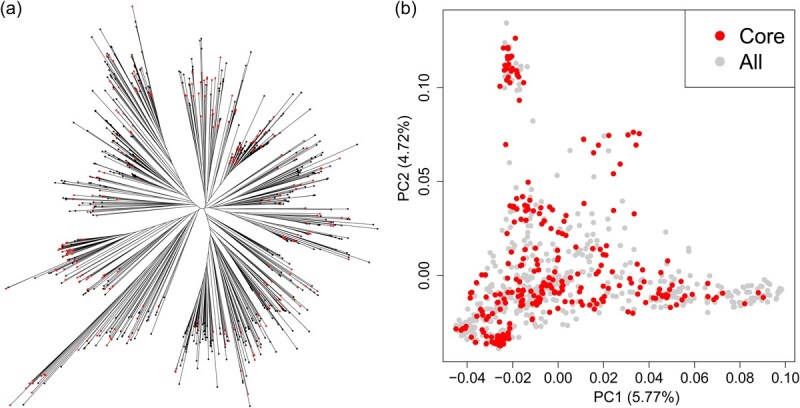
Development and evaluation of the lettuce core collection. (a) Phylogeny of 811 lettuce accessions. (b) PCA of 811 lettuce accessions. The clear separation of the core collection validated its utility as a distinct and structured group for downstream genetic studies. Red dots represent the accessions in 268 core collection lettuce; gray dots represent the accessions not in the core collection.

Whole-genome resequencing of 811 lettuce accessions at 1x depth generated 14 million SNPs, providing a high-resolution genomic landscape of global lettuce diversity (Table S1). Our main objective of developing a core collection from the 811 lettuce accessions is to provide the community with a subset of representative lettuce accessions, which can be used for future GWAS, quantitative trait locus (QTL) mapping, marker development, and gene cloning studies. The selected core collection would have a reasonable size (~300 accessions) and capture largely the allelic diversity of the entire collection, and also include accessions with some important agronomic traits. To develop this core collection, we integrated the genomic information with detailed agronomic traits information. Based on this combined selection, a core set of 268 accessions was selected from the 811 lettuce accessions, capturing 99.4% of the entire allelic diversity from the entire germplasm collection (Table S2). These core accessions were selected not only for their genetic variability but also for their diverse phenotypic characteristics, making them highly valuable for further research and breeding applications. Phylogenetic and principal component analysis (PCA) of these 268 accessions in the core collection (Fig. 1a and b) showed the nearly identical patterns to those of the ~800 accessions.

**Figure 2:**
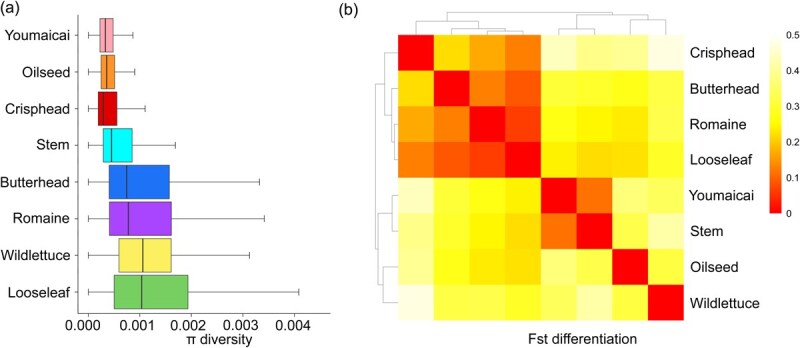
Genetic diversity in 268 core collection lettuce accessions. (a) Nucleotide diversity (π) of the lettuce populations. The statistics were calculated with 10-kb windows along the genome. (b) Pairwise differentiation *F*_st_. The heatmap represents averaged *F*_st_ with 10-kb windows along genome calculating pairwise *F*_st_ values for different crop types.

**Table 1:** The fixation index (*F*_ST_) and nucleotide diversity (π) of lettuce horticultural types

*F*_st_	Butterhead	Crisphead	Looseleaf	Oilseed	Romaine	Stem	Wild
Crisphead	0.174						
Looseleaf	0.069	0.092					
Oilseed	0.209	0.297	0.139				
Romaine	0.112	0.123	0.050	0.159			
Stem	0.278	0.334	0.207	0.286	0.235		
Wild	0.400	0.505	0.374	0.337	0.405	0.455	
Youmaicai	0.233	0.327	0.154	0.351	0.189	0.032	0.341
π	**1.01 × 10** ^**−3**^	**0.63 × 10** ^**−3**^	**1.21 × 10** ^**−3**^	**0.35 × 10** ^**−3**^	**0.97 × 10** ^**−3**^	**0.57 × 10** ^**−3**^	**1.16 × 10** ^**−3**^

π and *F*_st_ were calculated in a 100-kb sliding window with a 10-kb step using 7.15 M SNPs.

### Deeply resequencing and genetic diversity analysis of the lettuce core collection

To assess genomic diversity, the 268 core collection accessions (*L. sativa*) were deeply resequenced at ~5x coverage, alongside four wild lettuces (*Lactuca serriola*) resequenced by BGI-Shenzhen [3]. These accessions were geographically distributed across 29 countries, with the majority originating from China (110), the USA (49), Netherlands (35), and Turkey (20) (Table S2). Nineteen stem and three youmaicai were specifically selected from major producing areas in China. Approximately 27 billion paired-end raw reads (150 bp) were generated, with an average mapping rate of ~97.1% to the reference genome (Table S3). After applying basic filtering criteria (see Methods), a total of 7.15 million high-quality SNPs were retained, with minor allele frequency (MAF) >0.05, a maximum missing rate <0.2, and exclusively biallelic sites. Nucleotide diversity (π) decreased from 1.16 × 10^−3^ in wild relatives to 0.355 × 10^−4^ in oilseed and 0.331 × 10^−4^ in youmaicai (Fig. 2a, Table 1). This finding suggested that more than half of genetic diversity was lost during continuous cultivation and selection of lettuce cultivars from *L. serriola*, consistent with the previous studies [2, 3]. The pairwise genome-wide fixation index (*F*_st_) revealed higher levels of genetic differentiation between wild and cultivated crop types (Fig. 2b). In contrast, looseleaf presented high levels of genetic diversity while low levels of genetic differentiation among populations of cultivar lettuce (Fig. 2b, Table 1). However, youmaicai displayed the lowest *F*_st_ (0.032) relative to stem lettuce, which meant it had not differentiated from stem. The low genetic differentiation of youmaicai from stem was largely attributed to gene flow and restricted geographic adaptation.

### Phylogenetic relationships and population structure of lettuce core collection

**Figure 3:**
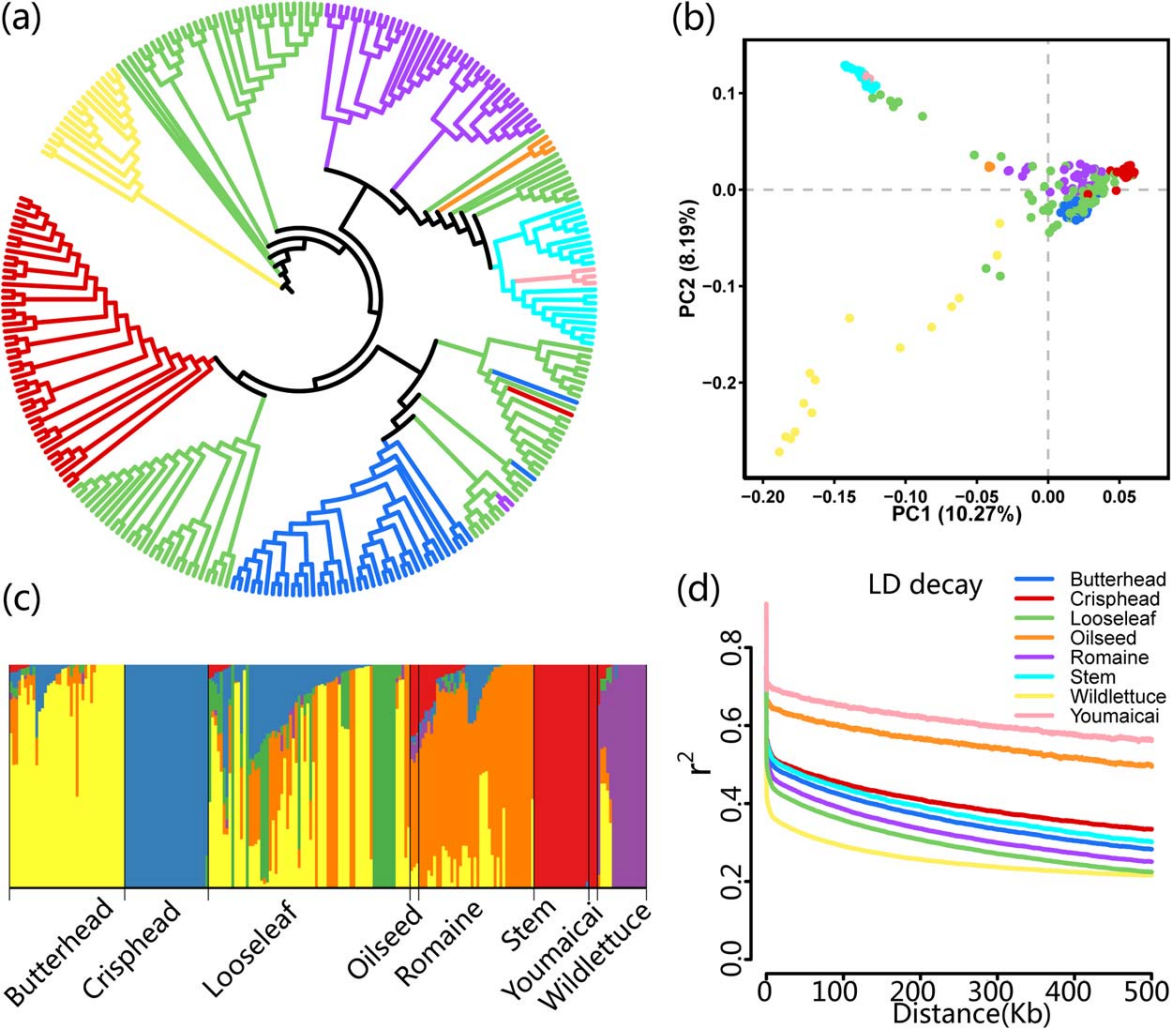
Phylogeny and population structure of 268 core collection lettuce accessions in eight horticultural types. (a) NJ tree based on a whole-genome filtered high-quality SNP dataset. (b) PCA plot of the first two PCs (PC1 and PC2). (c) Population structure of major horticultural types estimated by ADMIXTURE with the optimum number *K* = 6. Each color represents one ancestral population. Each colored segment in the bar represents the proportion of the ancestral relationship. (d) Decay of linkage disequilibrium in lettuce genome for wild and cultivars. Eight horticultural types are denoted by the lines in the color scale in (d).

To elucidate genetic relationships, phylogeny and population structure analyses were performed on the 268 lettuce core accessions. A neighbor-joining (NJ) tree, corroborated by a previous study, resolved that all cultivars formed five major clades and separated from wild relatives forming a monophyletic outgroup (Fig. 3a), consistent with horticultural classifications based on morphological architecture [2]. However, looseleaf cultivars exhibited broad dispersion across clades, complicating inferences regarding progenitor and derivative relationships [2, 3]. Notably, stem lettuce clustered within the romaine clade, supporting hypothesis of its domestication from romaine, reflecting a long historical lineage [2]. The primitive oilseed type further clarified evolutionary process, reflecting its historical role as a transitional form selected for seed oil production. Genetic proximity between youmaicai and stem lettuce suggested shared ancestry rather than independent classification. PCA revealed substantial genetic divergence among horticultural types, with PC1 and PC2 explaining 10.27% and 8.19% of the total variance, respectively (Fig. 3b). Population structure analysis using ADMIXTURE identified six ancestral subpopulations at *K* = 6, with no distinct clusters emerging for oilseed or youmaicai at higher *K* values (*K* = 7 or 8) (Fig. 3c). Linkage disequilibrium (LD) decay was measured by physical distance, which was estimated to be 220 kb on the whole (Fig. 3d). The analysis of population variance indicated a high level of genetic diversity and significant population differentiation.

### Phenotypic variations of leaf development traits across the lettuce core collection

**Figure 4:**
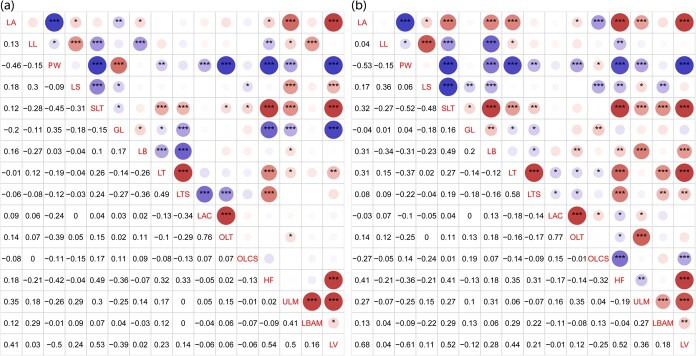
Correlation matrix of interrelationships among multiple leaf traits. (a) and (b) The Pearson correlation coefficients showed the strength of trait–trait relationships in the lower triangle between the phenotypic data collected in 2020 and 2021, respectively. Each trait is represented along the diagonal position, including LA, LL, PW, LS, SLT, GL, LB, LT, LTS, LAC, OLT, OLCS, HF, ULM, LBAM, and LV, in the 268 lettuce population. A threshold of marked pairwise correlations showed the levels of the correlation significance in the upper triangle, with ^*^, ^**^, and ^***^ depicted at *p* < 0.05, < 0.01, and < 0.001, respectively. Red indicates positive correlation while blue signifies negative correlation.

The core lettuce accessions, along with the full panel of 811 accessions, exhibited extensive phenotypic diversity in leaf morphology and developmental traits. Leaf structural and functional characteristics, critical for yield and quality in cultivated lettuce, were evaluated during stable autumn growing seasons in 2020 and 2021. Sixteen leaf morphology traits were quantified, including leaf attitude, leaf tip shape, anthocyanin coloration, thickness, blistering, plant width, glossiness, and outer leaf pigmentation head formation, etc (Table S4 and S5). The frequency distribution of each trait presented the range, central tendency, and variability of the traits across the 268 core accessions (Fig. S1). The boxplot showed the range and variability of each trait, such as plant width (PW) and leaf shape (LS) had wide distributions, indicating high diversity, while others may be more constrained. The majority of lettuce exhibited no leaf lobes of margin and leaf anthocyanin coloration (Fig. S1). These less common traits may confer a selective advantage in the studied environment, possibly due to their role in reducing water loss or enhancing light capture. Broad-sense heritability (H²) estimates for the 16 assessed morphological traits revealed a strong genetic basis for most characteristics in both years (Table S6). The majority of traits (three quarters) exhibited high heritability (H² > 0.80), such as leaf anthocyanin coloration (LAC: H² = 0.97), outer leaf color (OLC: H² = 0.96), shape of leaf tip (SLT: H² = 0.95), PW: H² = 0.94, head formation (HF: H² = 0.93), LS: H² = 0.93, and leaf venation (LV: H² = 0.93). Traits including leaf lobes of margin (LL), leaf attitude (LA), leaf blistering (LB), and undulation of leaf margin (ULM) also showed high heritability (H² = 0.85 - 0.92). Moderate heritability estimates (H² = 0.68 - 0.72) were observed for glossiness of leaf (GL), leaf blade incision in apical margin (LBAM), leaf texture (LT), outer leaf color intensity (OLCS), and leaf thickness (LTS), indicating a relatively greater influence of environmental factors on the phenotypic variation of these specific traits. These results highlight the significant potential for genetic improvement through phenotypic selection, particularly for traits with high H². The ANOVA analysis confirmed significant morphological differentiation among eight horticultural types across both years (Table S7 and S8). Traits such as HF and LV exhibited stable inter-group rankings, underscoring strong genetic control (supported by high heritability estimates, H² > 0.90). In contrast, LBAM and OLCS with lower heritability estimates showed greater environmental modulation. The distinct profiles of groups like Oilseed (minimal trait expression) and Youmaicai (extreme leaf shape/width) highlight their unique genetic backgrounds. These results validated the use of these traits for lettuce classification and breeding. Shannon-Wiener diversity index (H') for qualitative traits ranged from 0.40 to 2.15 (Table 2). The results indicated that eight traits—LTS, LS, LB, PW, GL, OLC, and OLCS—displayed high diversity (H′ > 1.00) across both years, while leaf morphology met this threshold in only 1 year, reflecting significant interaccession variation. The multivariate analysis of leaf traits revealed strong correlations among key morphological features (Fig. 4). The OLC showed significant positive associations with LAC (*r*^2^ = 0.76 in 2020; *r*^2^ = 0.77 in 2021). Conversely, the PW was negatively associated with most leaf traits, such as with LV (*r*^2^ = −0.61) and LA (*r*^2^ = −0.53), while LA exhibited positive correlations with LV (*r*^2^ = 0.68) in 2021. These interconnected traits likely reflect coordinated developmental pathways influencing leaf architecture.

**Table 2:** The distribution frequencies for ranking leaf characteristics of different types and diversity indexes of the analyzed accessions

Traits	1	2	3	4	5	6	7	8	9	10	H′
Shape of leaf tip 2020	10.99	24.61	64.40	0.00	0.00	0.00	0.00	0.00	0.00	0.00	0.87
Leaf thickness 2020	26.18	45.03	28.80	0.00	0.00	0.00	0.00	0.00	0.00	0.00	1.07
Leaf attitude 2020	25.79	0.00	63.16	11.05	0.00	0.00	0.00	0.00	0.00	0.00	0.88
Undulation of leaf margin 2020	0.00	0.00	61.78	25.13	13.09	0.00	0.00	0.00	0.00	0.00	0.91
Leaf shape 2020	9.42	16.75	5.76	13.61	15.18	2.62	14.14	10.99	1.57	9.95	2.15
Leaf blistering 2020	36.13	0.00	34.03	0.00	15.18	0.00	14.66	0.00	0.00	0.00	1.30
Plant width 2020	6.81	0.00	13.61	0.00	37.70	0.00	28.27	0.00	13.61	0.00	1.45
Leaf blade incision in apical margin 2020	36.65	0.00	0.00	0.00	0.00	0.00	0.00	0.00	63.35	0.00	0.66
Glossiness of leaf 2020	27.75	35.08	36.65	0.52	0.00	0.00	0.00	0.00	0.00	0.00	1.12
Outer leaf color 2020	42.93	22.51	15.71	18.85	0.00	0.00	0.00	0.00	0.00	0.00	1.30
Leaf venation 2020	50.79	49.21	0.00	0.00	0.00	0.00	0.00	0.00	0.00	0.00	0.69
Outer leaf color intensity 2020	0.00	0.00	29.32	0.00	36.65	0.00	34.03	0.00	0.00	0.00	1.09
Leaf anthocyanin coloration 2020	66.49	0.00	0.00	0.00	0.00	0.00	0.00	0.00	33.51	0.00	0.64
Head formation 2020	58.64	17.80	23.56	0.00	0.00	0.00	0.00	0.00	0.00	0.00	0.96
Leaf texture 2020	85.86	14.14	0.00	0.00	0.00	0.00	0.00	0.00	0.00	0.00	0.41
Leaf lobes of margin 2020	85.86	7.33	6.81	0.00	0.00	0.00	0.00	0.00	0.00	0.00	0.51
Shape of leaf tip 2021	12.62	13.59	73.79	0.00	0.00	0.00	0.00	0.00	0.00	0.00	0.76
Leaf thickness 2021	36.89	29.61	33.50	0.00	0.00	0.00	0.00	0.00	0.00	0.00	1.09
Leaf attitude 2021	21.84	0.00	27.18	50.97	0.00	0.00	0.00	0.00	0.00	0.00	1.03
Undulation of leaf margin 2021	0.00	0.00	75.73	17.48	6.80	0.00	0.00	0.00	0.00	0.00	0.70
Leaf shape 2021	14.08	10.68	6.31	17.96	14.08	4.85	5.34	12.62	0.00	14.08	2.11
Leaf blistering 2021	18.93	0.00	33.98	0.00	22.82	0.00	14.08	0.00	10.19	0.00	1.53
Plant width 2021	7.28	0.00	19.90	0.00	41.75	0.00	24.27	0.00	6.80	0.00	1.40
Leaf blade incision in apical margin 2021	32.04	0.00	0.00	0.00	0.00	0.00	0.00	0.00	67.96	0.00	0.63
Glossiness of leaf 2021	28.64	34.47	36.89	0.00	0.00	0.00	0.00	0.00	0.00	0.00	1.09
Outer leaf color 2021	60.98	13.66	8.78	16.59	0.00	0.00	0.00	0.00	0.00	0.00	1.09
Leaf venation 2021	45.63	54.37	0.00	0.00	0.00	0.00	0.00	0.00	0.00	0.00	0.69
Outer leaf color intensity 2021	0.00	0.00	32.68	0.00	45.37	0.00	21.95	0.00	0.00	0.00	1.06
Leaf anthocyanin coloration 2021	69.42	0.00	0.00	0.00	0.00	0.00	0.00	0.00	30.58	0.00	0.62
Head formation 2021	55.83	11.17	32.52	0.00	0.49	0.00	0.00	0.00	0.00	0.00	0.96
Leaf texture 2021	69.42	30.58	0.00	0.00	0.00	0.00	0.00	0.00	0.00	0.00	0.62
Leaf lobes of margin 2021	89.32	8.25	2.43	0.00	0.00	0.00	0.00	0.00	0.00	0.00	0.40

H′ represents Shannon–Wiener diversity index.

To group together horticultural types that are phenotypically similar across all measured traits, we performed hierarchical clustering on the 16 phenotypic data by a distance matrix using Euclidean distance. The phylogenetic-like relationships based on phenotype revealed that horticultural types clustered closely together (e.g., stem clustered with the romaine) shared very similar morphological characteristics (Fig. S2a and b). This makes biological sense as they may have shared ancestry or convergent evolution for certain traits. Crisphead and oilseed far apart on the tree were phenotypically the most distinct, due to crisphead forms tight and dense heads, while oilseed types are selected for seed production, not leaf morphology. Moreover, PC1 and PC2 accounted for approximately 40% of the variance in leaf traits (Fig. S2c and d), indicated phenotypically distinct was greater. This was a strong visual confirmation of the measured traits effectively discriminated between the major lettuce types. The phylogenetic-like relationships and PCA of leaf traits, similar to genetic analysis, indicated significant correlations existed among the lettuce core collection germplasms. These results revealed the key agronomic traits were highly heritable, and validated that the phenotypic data captured known biological relationships and differences.

### Genome-wide association analyses for agronomic traits in the lettuce core accession

**Figure 5:**
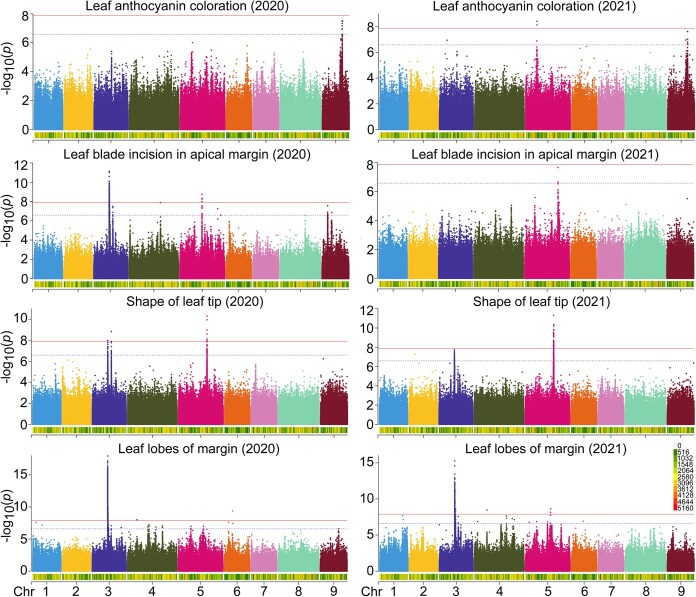
Significant marker–trait associations for lettuce leaf traits in 2020 and 2021. QTLs were detected through genome-wide association analysis using an MLM model in GAPIT3 in the lettuce genome. The *y*-axis in each graph represents −log_10_(*p*) for the *P-*value from genotypic associations, while the *x*-axis represents the marker density on chromosomal locations. The marker density along the chromosomes are denoted by the squares in the color scale in the bottom right corner.

Genome-wide association studies were performed using a mixed linear model (MLM) model implemented in GAPIT3 to identify QTLs associated with leaf morphology and pigmentation. A final set of 3 842 327 high-quality SNPs, derived from 268 lettuce accessions, was analyzed across two independent growing seasons (2020 and 2021). Eleven QTLs were consistently detected for four traits at a genome-wide significance threshold (−log10(*p*) ≥ 7.0), with lead SNPs localized to defined genomic intervals (Table 3). The quantile-quantile (Q-Q) plots displayed a strong deviation from the null distribution, and the inflation (λ) values ranged from 0.61 to 0.72, indicating slight deflation rather than inflation, which could be ascribed to the strong association observed within the leaf traits (Fig. S3). For LAC, two QTLs were detected on chromosome 5 (*qLAC5.1*) and 9 (*qLAC9*). Moreover, 9 additional QTLs were identified on chromosomes 3 and 5 for leaf shape, margin, and tip morphology (Table 3). Candidate genes of these QTLs were annotated in the supplementary Table S9–S12, and all significant SNPs were associated with genes listing in Table S13. For the remaining 12 traits, the GWAS results suggested no significant associations were detected across both 2020 and 2021, except for leaf shape (2020) and outer leaf color (2021), and Q-Q plots showed that the distribution of the most significant associations was in the lower tail (Fig. S4). A multi-environment GWAS was performed to distinguish stable genetic effects from year-specific interactions. We calculated Best Linear Unbiased Predictions (BLUPs) for the combined 2020 and 2021 phenotypic datasets to account for environmental variability. In contrast, the multi-environment GWAS using BLUP values identified stable, consensus QTLs for all four traits (Fig. S5), which were consistently detected across both years (Fig. 5), indicating a stronger genetic component for their phenotypic diversity. Both the year-specific and multi-environment results highlight the identified QTLs with robust, environment-independent genetic control in shaping phenotypic variation over these four traits of lettuce leaf. Furthermore, the comparison of GWAS results from three additional models, including CMLM (Compressed Mixed Linear Model), GLM (General Linear Model), and FarmCPU (Fixed and Random Model Circulating Probability Unification), revealed that the most significant QTLs were robustly associated by multiple models (Fig. S6). While MLM provided a balanced approach for our dataset, and the consistency across models strengthened the credibility of the identified loci.

**Table 3:** Summary of GWAS loci from four leaf traits in 2020 and 2021.

Loci	QTLs	Traits	Chr	Lead SNP	-log10(p)	QTL Interval
1	qLAC9	LAC_2020	9	9:151817808	7.86	151286710-152765187
2	qLAC9	LAC_2021	9	9:151715491	7.62	151715491-151715513
3	qLAC5	LAC_2021	5	5:84586279	8.42	84586279-85956385
4	qLBAM3	LBAM_2020	3	3:117309548	10.12	117297748-119386680
5	qLBAM5	LBAM_2020	5	5:167648220	8.93	167285278-167648220
6	qSLT3.1	SLT_2020	3	3:117451582	7.46	117349004-117664300
7	qSLT3.2	SLT_2020	3	3:144782677	8.41	144777531-144785505
8	qSLT5.1	SLT_2020	5	5:217056591	10.792	14476511-217654950
9	qSLT5.1	SLT_2021	5	5:215250470	11.322	14652707-217654950
10	qSLT5.2	SLT_2021	3	3:117389400	7.74	117368907-117713823
11	qLL3.1	LL_2020	3	3:117552695	18.19	116456690-119386680
12	qLL3.1	LL_2021	3	3:117368907	15.26	117297501-119386031
13	qLL3.2	LL_2021	3	3:123472728	11.89	123404332-123497738
14	qLL5	LL_2021	5	5:191612919	8.62	191338767-191612919

Note: Chr, chromosome; MAF, minor allele frequency; P-value, the significance was estimated by GWAS in 2020 and 2021, respectively; Each trait is represented as LBAM, LL, SLT, and LAC.

**Figure 6:**
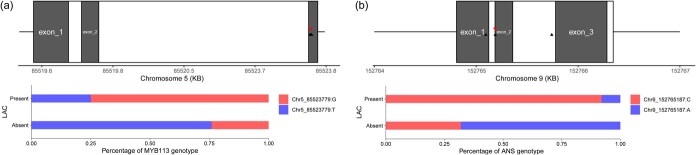
The potential causal genes were identified from phenotypes and candidate genes of GWAS. (a) and (b) The positions of candidate variations are indicated by a black triangle along the representative *MYB113* and *ANS* genes. The red arrows represent the position of the key variants in Chr. 5:85523779 (missense variant) and Chr. 9:152765187 (stop lost) from causal genes of LAC. Histograms underneath the causal gene structure represent the percentage of genotypes of *MYB113* and *ANS* in 191 *L. sativa* accessions.

Candidate gene analysis within QTL intervals revealed 28 genes associated with LAC, including *MYB113* (*Myb domain protein 113*) and *ANS* (*Anthocyanidin synthase*), both implicated in flavonoid biosynthesis [2]. Functional variants were identified in these loci: a missense mutation in *MYB113* (Chr5:85523779) and a stop-loss variant in *ANS* (Chr9:152765187) (Fig. 6). These variants were predominantly enriched in accessions exhibiting anthocyanin pigmentation (*p* < 0.001), suggesting causal roles in leaf color regulation. The *qLAC5* interval also harbored three disease resistance genes (*RPM1*), indicating potential pleiotropy between pigmentation and pathogen response pathways. Six leaf shape QTLs (*qLBAM3*, *qSLT3.1*, *qSLT3.2*, *qLL3.1*, *qLL3.2*) clustered within a 27-Mb region on chromosome 3 (117–144 Mb), influencing subtle margin serration. A seventh QTL (*qSLT5.1*), localized to 214–217 Mb on chromosome 5, was associated with leaf tip variation. These QTLs revealed that the coordinated activity of genes from various pathways could be integrated into regulatory network for leaf architectural traits in lettuce.

### Synergistic improvement of anthocyanin content and leaf morphology through genomic design-based breeding

**Figure 7:**
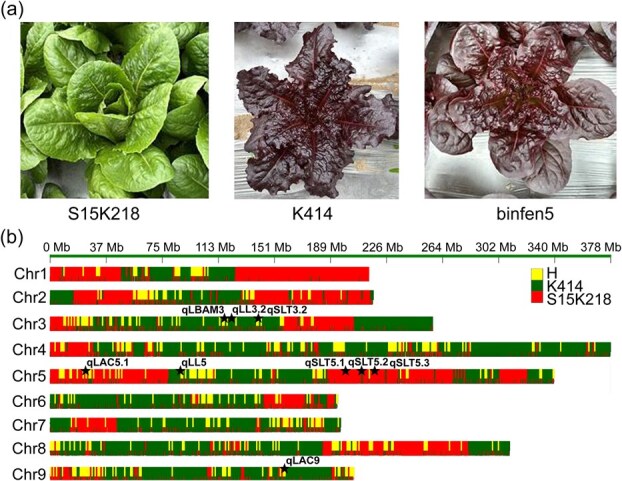
The lettuce variety through genomic design breeding. (a) The phenotype of two parents and offspring. (b) The fingerprint of the offspring from K414 $\times $ S15K218. The black stars indicated the QTL intervals identified using GWAS in this study.

Anthocyanins are key polyphenolic compounds that contribute significantly to color, flavor, and antioxidant properties in many crops, and enhance both nutritional value and consumer appeal. Their strong antioxidant activity mitigates oxidative stress, potentially reducing risks of chronic diseases. Leveraging GWAS-derived QTLs, we investigated the genetic basis of anthocyanin accumulation and leaf morphology to design improved lettuce cultivars. By examining a comprehensive sequenced panel of accessions, we focused on identifying two high-anthocyanin alleles that could be combined to maximize anthocyanin production. Our initial scan revealed that several accessions harbored favorable alleles linked to anthocyanin accumulation on chromosome 5 and chromosome 9. Accession K414, harboring favorable high-anthocyanin alleles at both loci, was crossed with S15K218 (elite leaf morphology) to generate a biparental population. Over five successive generations of careful selection and phenotypic evaluation, we successfully developed a novel high-anthocyanin variety binfen5. The new variety not only demonstrated an enhanced anthocyanin content but also maintained an excellent leaf morphology closely resembling that of the original S15K218 accession, ensuring that desirable esthetic traits were preserved (Fig. 7a and Fig. S7).

Further genetic analysis was conducted using a fingerprint mapping approach. The fingerprint map of the developed variety, designated as ‘binfen5’, revealed that the QTLs associated with anthocyanin content had been successfully introgressed from the parent accessions K414, while five of the rest of eight-leaf morphology QTLs were originated from S15K218S (Fig. 7b). This analysis provided us with a robust dataset from which we could select the most promising candidates for further breeding work and the potential of GWAS-based genomic design breeding as an efficient method for developing superior horticultural varieties. By genomic selection and combining alleles with complementary effects, our approach not only improves the nutritional profile by enhancing anthocyanin content but also boosts the market appeal of lettuce.

## Discussion

Our analysis identified specific genomic regions with substantial effects on leaf traits, demonstrating associations between morphological variation and genetic architecture, thereby enhancing understanding of leaf trait regulation in lettuce. Moreover, the integration of genomic design breeding into lettuce improvement presents a challenge for translating genetic discoveries into agricultural applications.

### The genetic diversity of the lettuce core accession

Leaf morphological traits exhibit significant variation as adaptations to multiple climate gradients in lettuce. The lettuce core accessions (from 29 countries) present an opportunity to address genetic diversity by incorporating field data from multiple agroecological zones. Leaf appearance and color are crucial traits influencing consumer preference. The typical green pigmentation diversified during domestication, with red leaf varieties emerging through flavonoid and anthocyanin accumulation [15]. Similarly, leaf architecture diversified into nonheading, loose-heading, and tight-heading morphotypes [16]. This study revealed higher diversity indices for leaf shape, plant width, and outer leaf color compared to other traits (Table 2). Continuous phenotypic distributions and significant correlation, such as between outer leaf color and anthocyanin content (Fig. 4), underscore the suitability of this population for genetic mapping. These trait correlations provide critical insights for breeding programs targeting desirable characteristics.

Advances in sequencing technology have enabled comprehensive studies of the genetic control of morphological patterns and resolution of genetic diversity among lettuce cultivars [17]. Phylogenetic analysis showed that horticultural types formed distinct monophyletic clades, except for looseleaf varieties (Fig. 3a). Wild lettuce accessions clustered into a single group with greater genetic diversity and divergence from cultivars (Fig. 3a and b), consistent with a single domestication origin followed by human-mediated diversification [2, 3]. Looseleaf accessions exhibited genetic admixture within cultivated clades, likely reflecting intercrossing and localized breeding. Population differentiation between stem and leaf lettuce suggests independent domestication events, probably originating in China and propagated into stem-use (e.g. youmaicai) and leaf-use types. Notably, Egyptian oilseed accessions clustered closely with romaine varieties, while stem lettuce showed ancestral ties to romaine and looseleaf types, supporting historical dispersal via trade routes like the Silk Road. Consequently, different diets in different countries have led to discrimination and diversity of lettuce cultivars.

### Genetic discoveries and mechanistic insights for lettuce leaf traits

High genetic heterogeneity and phenotypic variation were enabled by genome-wide association studies to elucidate leaf trait regulation [18]. Our research on leafy head provided insights into molecular mechanisms underlying changes in leaf architecture. The measurement of leaf quality included the important characteristics, appearance, color, texture, and flavor [16]. The GWAS model justification and combined phenotypic analyses revealed stable genetic associations for LAC, LBAM, SLT, and LL consistent across different environments. The color spectrum in lettuce cultivars, from green and yellow to deep red, was attributed to varying concentrations of chlorophyll and anthocyanin in the leaves [19]. Anthocyanin, polyphenols, and flavonoids have been reported to play significant antioxidant roles in lettuce [20]. These metabolites are often regarded as health-beneficial nutrients, which adjusted consumer preference for lettuce types [21]. Two candidate genes, *ANS* (*Anthocyanin synthase*) and *MYB113* (*MYB domain protein 113*), were identified in our mapping analysis of LAC, providing new evidence for the previously established flavonoid biosynthesis pathway [2].

Dissection of genetic loci for leaf morphology traits could accelerate the breeding of high-quality lettuce varieties. In this study, the key variants of *MYB113* and *ANS* presented predominantly in the present anthocyanin coloration group, which will be valuable for breeding. Leaf color control genes have been reported to undergo disruptive selection since domestication, such as *R2R3-MYB* and *R3-MYB* similar to *MYB113* [22]. Leaf morphology consists of wide variable traits, which enable the adaptation of plants to different natural environments and habitats [23]. The leaf edges were smooth, undulating, pustule, lobes, incision, and other shapes, forming an incredible diversity of leaf shapes [23]. Significant genetic factors were mapping to be interesting candidate genes to govern the elaboration of leaf margins (Fig. 5, and Fig. S5), for example *BGL2* and *GRV2*, which were also associated with leaf shape. Based on the correlation and coordination of multitraits, a few common QTLs among blade incision, lobes, and shape of leaf tip, which were closely related in phenotype correlation and genetic mapping. This suggested that these traits could be the core indicators to characterize leaf morphology. Plants with highly lobed leaves improved canopy structure and achieved significantly greater biomass than that with less lobed leaves [24]. The LAC was independent of them and its regulatory mechanisms of core genes are network-dependent [2, 25]. Considering the importance of these phenotypic traits, the integration of phenotype correlation and genetic mapping is critical for understanding of the genetic architecture of leaf morphology. Although candidate genes were lacking biological verification, the detected QTLs would contribute to effectively assessing the genetic basis of leaf traits, such as known genes *AGL44*, *MYB113*, *MYC2*, and *ANT* [26–28]. This genetic study provides a better implication in pathway regulation for improving developmental control of the formation leaf traits.

### Genomic design breeding in lettuce field trial

To bridge the gap between genomic discovery and agricultural impact, a challenge underscored in complementary field validation [29]. Allelic variation analysis in this study highlighted favorable alleles for anthocyanin accumulation, enabling marker-assisted selection of high-pigmentation lines with stable phenotypes across environments. Based on the above results from both the year-specific and multi-environment GWAS, we conducted allelic variation analysis and identified two favorable alleles, one on each locus, which consistently resulted in elevated anthocyanin levels across diverse germplasm. By adopting a hybrid approach, we successfully selected high-anthocyanin varieties with aggregating these favorable alleles through marker-assisted selection (MAS). The binfen5 exhibited significantly higher pigmentation and leaf morphology throughout multiple growth stages, demonstrating both robustness and consistency (Fig. S7). It ensured that genomic insights translate into tangible improvements in lettuce visual appeal and health benefits that combine controlled-environment precision with field-based robustness.

## Conclusion

Our study established a core collection of 268 lettuce accessions that preserves 99.4% of global genetic diversity, providing a valuable resource for breeding and evolutionary studies. Population structure analyses clarified the close evolutionary relationship between stem lettuce and romaine, revealed extensive introgression from looseleaf types, and highlighted the genetic diversity maintained within each subgroup. By leveraging genomic tools to optimize allele combinations, we demonstrated the potential of genomic design breeding to effectively balance nutritional and aesthetic traits. In particular, QTLs identified through GWAS enabled the development of a high-anthocyanin variety with favorable leaf morphology. These results provide both fundamental insights and practical strategies for accelerating lettuce breeding and enhancing crop quality. Future efforts should expand this core collection to dissect additional agronomic traits and validate candidate genes through functional studies.

## Materials and methods

### Core collection selection and phenotypic evaluation

A diverse collection of 811 lettuce accessions, including cultivars, landraces, and wild relatives, was obtained from the Shanghai Agrobiological Gene Center [30]. Young leaves from each accession were harvested and genomic DNA was isolated using the cetyltrimethylammonium bromide (CTAB) method. Sequencing libraries were prepared with an average insert size of 400 bp and sequenced on a DNBSEQ™ sequencing technology platform to generate paired-end reads (2 × 150 bp). Each accession yielded ~3 Gb of raw data (Table S1). Quality control of the sequencing data was conducted by using AdapterRemoval (V2) [31] to trim low-quality regions (average phred score <20) and remove adapter-contaminated or short reads (<50 bp)^.^ Filtered reads were mapped to the *Lactuca sativa* cv. Salinas V8 reference genome (CoGe ID: gid_28333) using BWA-MEM (V0.7.12) with the default parameters. Aligned reads were sorted and converted to BAM files using SAMtools (V1.9), and duplicates were marked using MarkDuplicates implemented in Picard (V2.24.0) (http://broadinstitute.github.io/picard/). Genomic regions around indels were realigned using RealignerTargetCreator and IndelRealigner packages in the Genome Analysis Toolkit (GATK, V4.1.9.0) [32]. Variants were called for each accession using HaplotypeCaller in -ERC GVCF mode, followed by joint genotyping of all accessions with individual gVCF using GenotypeGVFs. SNPs were filtered using GATK’s VariantFiltration with stringent thresholds: QD < 2.0||FS > 60.0||MQ < 40.0||MQRankSum < −12.5||ReadPosRankSum < −8.0. Only biallelic SNPs with an MAF ≥0.01 were retained. To minimize selection bias, 37 537 SNPs at 4-fold degenerate synonymous sites were used for core collection analysis.

To establish a representative core subset, we first performed hierarchical clustering based on pairwise genetic distances calculated from the SNP dataset. Then, we compiled candidate accessions used in breeding programs that carried elite alleles for key traits, including heat tolerance, high nutrition content, bolting resistant et.al. Additionally, the geographic origins and horticultural types were used to select the representation of the core collection (Table S2). We then evaluated the genetic diversity using GenoCore (V1.0) [33], with parameters –d 0.01% (minimum distance threshold), −cv 100% (coverage of allelic diversity). By combining the GenoCore output with the breeding priority accessions, we obtained the final core collection containing 268 lettuce accessions. Population structure was assessed using PCA (GCTA V1.93.2) [34] and phylogenetic analysis using FastTree2 (V2.1.11) [35] under the GTR + CAT model. Then, the core collection accessions were resequenced to an average depth of 5x with 15 Gb per sample. Data were reprocessed using the same pipeline (BWA-MEM, GATK) to ensure consistency with the initial 811 accessions. The identified core collection originated from a broad geographic distribution spanning 29 countries, and detailed metadata for each accession were provided in Table S2, including accession names, countries of origin, and cultivated types. Biallelic SNPs were retained for the subsequent analysis with the criteria: a missing rate <10% and an MAF <0.05. After the stringent filtering, the 7 156 445 high-confidence SNPs were used for population genetic inferences, and annotated using snpEFF (V5.1) [36] based on the *L. sativa* cv. Salinas V8 genome annotation.

Field experiments were conducted at the Shanghai Agrobiological Gene Center (Shanghai, China) during the autumn growing seasons of 2020 and 2021. A randomized complete block design (RCBD) with three biological replicates was implemented, with individual plants spaced at 25-cm intervals to minimize environmental variation. Sixteen agronomic traits (detailed in Table 4) critical to lettuce growth and development were evaluated in duplicate across both seasons, adhering to the test of distinctiveness, uniformity, and stability (DUS) of lettuce approved by UPOV guidelines (TG/13/11 Rev. 3) [37, 38]. Traits were categorized as follows: undulation of leaf margin (ULM), leaf blade incision in apical margin (LBAM), head formation (HF), leaf attitude (LA), leaf lobes of margin (LL), shape of leaf tip (SLT), leaf shape (LS), leaf texture (LT), leaf thickness (LTS), leaf blistering (LB), plant width (PW), glossiness of leaf (GL), outer leaf color (OLT), leaf venation (LV), outer leaf color intensity (OLCS), and LAC. Quantitative measurements were mainly conducted using visual assessment by observation of individual/group plants on the basis of the expert's judgment. Data were collected from at least 15 plants per accession to ensure statistical robustness, available in Tables S4 and S5.

**Table 4:** Description of lettuce characters

Traits	Qualitative traits and assignment criterion for characteristic classification									
	1	2	3	4	5	6	7	8	9	10
Shape of leaf tip	Acute	Obtuse	Rotundate							
Leaf thickness	Thin	Medium	Thick							
Leaf attitude	Erect		Semi-erect		Horizontal					
Undulation of leaf margin	Absent or extremely weak		Weak		Medium		Strong		Extremely strong	
Leaf shape	Narrow elliptic	Medium elliptic	Broad elliptic	Orbicular	Wide broad elliptic	Wide oblong	Obovate	Broad rhombic	Triangular	Lanceolate
Leaf blistering	Absent or extremely weak		Weak		Medium		Strong		Extremely strong	
Plant width	Extremely small		Small		Medium		Large		Extremely large	
Leaf blade incision in apical margin	Absent								Present	
Glossiness of leaf	Week	Medium	Strong							
Outer leaf color	Absent	Yellowish green	Grayish green	Red						
Leaf venation	Nonflaballate	Flaballate								
Outer leaf color intensity			Light		Medium		Dark			
Leaf anthocyanin coloration	Absent								Present	
Head formation	Nonheading	Loose heading	Tight heading							
Leaf texture	Soft	Crisp								
Leaf lobes of margin	Absent	Lobed	Parted							

The corresponding visualizations for frequency distributions were generated into histograms by R (v4.3.1). ANOVA analysis of variance was conducted by using R car packages to provide statistical support for leaf trait differences between groups. Broad-sense heritability was calculated by using a mixed model with R lme4 package. To visualize and quantify the structure of diversity within the core collection lettuce germplasms, the phylogenetic and PCA on phenotypic data were performed in R. The phenotypic distance matrix was calculated with Euclidean distance, and then performed hierarchical clustering using hclust function to construct the dendrogram. PCA plots were created with R prcomp function and ggplot package.

### Population genetics analysis using lettuce core collection

Genome-wide estimates of nucleotide diversity (π) and genetic differentiation (*F*_ST_) were calculated using VCFtools (V0.1.16) [39], with a sliding window approach (window size = 100 kb, step size = 10 kb). LD was assessed using PopLDdecay (V3.31, https://github.com/BGI-shenzhen/PopLDdecay), with parameters: –MaxDist 5000 –MAF 0 –Het 1 –Miss 1. The LD decay rate was defined as the physical distance at which the genome-wide average *r*^2^ declined to half its maximum value, following methodologies established in cucurbit crops [11]. An NJ tree was constructed using PHYLIP (V3.697) to elucidate phylogenetic relationships [40]. The wild relative *L. serriola* served as the outgroup. The NJ tree was visualized and annotated using the iTOL tree viewer (V6) (http://itol.embl.de). PCA was performed with Genome-wide Complex Trait Analysis (GCTA, V1.26.0) [34], and the first two eigenvectors were displayed in 2D plot to visualize genetic clustering. Population structure was inferred by using ADMIXTURE (V1.3.0) [41], with the number of genetic clusters (*K*) ranging from 2 to 10. The optimal K was determined by minimizing cross-validation (CV) error, and the most likely subgroups were identified. Based on prior evidence of irregular introgression in other horticultural types [2], admixed looseleaf accessions with the wild species were omitted to clarify domestication signals in population admixture analysis.

### Genome-wide association study of leaf traits

Association mapping for the 16 agronomic leaf traits was conducted using the compressed MLM implemented in GAPIT3 [42]. To minimize LD bias, SNPs were pruned via LD-based clumping (PLINK V1.90b parameters: –indep-pairwise 20 kb 5 0.2), retaining 3 842 327 SNPs with MAF >0.1 and missing rate <10%. The first three PCs were included as fixed effects to control for population stratification. Then, the genome-wide significance was determined using permutation-based threshold with –log10(1/3842327) = 6.585 (*P* < 2.6 × 10^−7^) and Bonferroni multiple test threshold with –log10(0.05/3842327) = 7.886 (adjusted *p*< 1.3 × 10^−8^) [43]. Given the conservative nature of Bonferroni correction, a compromised threshold (−log10(*p*) ≥ 7.0) was applied for candidate SNP prioritization. Significant SNPs were clustered into QTL regions if physical proximity was >200 kb between adjacent SNPs and the density was more than three significant SNPs per region. The most significant SNP per region was regarded as the lead SNP. Manhattan and Q-Q plots were generated via GAPIT3 to visualize associations. In addition to year-specific GWAS analyses, the best linear unbiased predictor (BLUP) values were calculated for a combined analyses of the 2020 and 2021 phenotypic datasets by R lme4 package. We performed multi-environment GWAS with the BLUP values as a phenotype to distinguish stable genetic effects from environment-specific interaction in GAPIT3.

### Construction of the fingerprinting map for hybrid population progeny

The core request is from genetic discovery to genomic design breeding, boosting rapid utilization of elite cultivars for the market drivers. Currently, in romaine lettuce, the dominant commercial varieties are green, with limited red options. Our initial goal was to develop a red romaine lettuce to fill this gap. During the development of the core lettuce germplasm collection, we identified an opportunity to enhance anthocyanin content (linked to health-promoting polyphenols) while maintaining elite morphology. The genetic analysis clued to parental selection strategy based on leaf morphology and QTLs for genomic design breeding. A high-anthocyanin accession K414 contains favorable alleles at two key loci, contributing deep red pigmentation and high polyphenol content; while an elite romaine line S15K218 ensures commercial viability with superior leaf morphology, crispness, and yield. The cross of K414 and S15K218 generated genetic diversity biparental population. To use SNPs to track high-anthocyanin alleles by MAS, we scanned generations of inbreeding and selection in massive populations. Over five successive generations, the desired combination of traits was fixed to create a uniform, stable inbred line binfen5. To confirm uniform appealing novelty across environments, DUS testing was performed in Shanghai, China, and processed to pass Shanghai’s official variety certification (Fig. S7a). Large-scale adoption of binfen5 by farmers demonstrates its robust performance in local production conditions and economic viability (Fig. S7b).

Parental SNPs (K414 and S15K218) were first extracted from the population variant matrix. Only SNPs meeting the following criteria were retained for downstream analysis: fixed homozygous genotypes in both parents and polymorphic divergence between parents. For each retained SNP locus, the genotype of progeny binfen5 was determined as male homozygous, female homozygous, or heterozygous. To reduce noise from individual SNP calls and to identify contiguous chromosomal segments inherited from each parent, we implemented a binning approach. SNPs were grouped into nonoverlapping bins of 1 Mb along each chromosome. Within each bin, the counts of male homozygous, female homozygous, and heterozygous genotypes were tallied across all informative SNPs. The genotype category with the highest frequency in a given bin was assigned as the bin genotype. The resulting fingerprinting (bin) map, depicting the parental inheritance of chromosomal segments and recombination breakpoints, was visualized using the RectChr software under default plotting parameters (https://github.com/hewm2008/RectChr).

## Supplementary Material

Web_Material_uhaf258
